# Ultrashort-T_2_* mapping at 7 tesla using an optimized pointwise encoding time reduction with radial acquisition (PETRA) sequence at standard and extended echo times

**DOI:** 10.1371/journal.pone.0310590

**Published:** 2025-04-17

**Authors:** Carly A. Lockard, Bruce M. Damon, Hacene Serrai

**Affiliations:** 1 Stephens Family Clinical Research Institute, Carle Health, Urbana, Illinois, United States of America; 2 Carle Illinois Advanced Imaging Center, Urbana, Illinois, United States of America; 3 Department of Bioengineering, University of Illinois at Urbana-Champaign, Urbana Illinois, United States of America; 4 Beckman Institute, University of Illinois at Urbana-Champaign, Urbana, Illinois, United States of America; 5 Department of Biomedical Engineering, Vanderbilt University, Nashville, Tennessee, United States of America; 6 Department of Radiology and Radiological Sciences, Vanderbilt University, Nashville, Tennessee, United States of America; 7 Carle Illinois College of Medicine, University of Illinois at Urbana-Champaign, Urbana Illinois, United States of America; Museo Storico della Fisica e Centro Studi e Ricerche Enrico Fermi, ITALY

## Abstract

Zero echo time (ZTE) sequences capture signal from tissues with extremely short T_2_* and are useful for qualitative and quantitative imaging of musculoskeletal tissues’ ultrashort-T_2_* components. One such sequence is Pointwise Encoding Time Reduction with Radial Acquisition (PETRA). While this sequence has shown promising results, it has undergone only limited testing at 7 tesla (T). The purpose of this work was to evaluate PETRA at 7T in its standard, commercially available form and with sequence code modifications to allow extended echo times for the purpose of performing ultrashort-T_2_* mapping. We acquired PETRA images of MnCl_2_ and collagen phantoms and of the knee in eight participants (5 for optimization and 3 for ultrashort-T_2_* mapping assessment; 5 male/3 female, 39 ± 11 years old). Images were acquired using a 1-transmit/28-receive-channel knee coil. Artifacts, signal, signal-to-noise ratio (SNR), ultrashort-T_2_*, the corresponding curve fit quality, and repeatability were assessed. In knee tissues, SNR was higher at TE = 0.07 msec than in a conventional-TE sequence (Dual-Echo Steady State with TE = 2.55 msec), with values of 68–337 for PETRA versus 16–30 for the same regions in the conventional-TE series. Acquisition of series for ultrashort-T_2_* maps was feasible at 1.50 mm isotropic acquisition resolution and TE ≤ 0.58 msec. Strong linear correlations were observed between relaxation rates (R_2_*) and MnCl_2_ concentration, and between signal and collagen concentration. Ultrashort-T_2_* signal decay curve fit *R*^2^ and repeatability were high for phantom and knee ultrashort-T_2_* <1 msec. PETRA imaging with minimal artifacts, high SNR, and scan time < 11 minutes was achieved at 7T at high (0.34 mm isotropic) resolution at TE = 0.07 msec and lower resolution (1.52 mm isotropic) at echo times ≤ 0.58 msec. Ultrashort-T_2_* mapping provided acceptable curve-fitting results for substances with sub-millisecond T_2_*.

## Introduction

Conventional magnetic resonance imaging (MRI) sequences provide limited visualization of tissues with short/ultrashort T_2_* values, due to their inability to reduce the echo times (TE) sufficiently to acquire signal from these tissues prior to signal decay. However, ultrashort echo-time (UTE) and zero echo-time (ZTE) MRI sequences are designed to acquire signal from these tissues, enabling both qualitative and quantitative evaluations. In the musculoskeletal system, UTE and ZTE imaging are particularly useful for studies of bone, tendon, calcified cartilage, meniscus, and other organs [1–11]. One ZTE sequence that has begun to be applied to these tissues is Pointwise Encoding Time Reduction with Radial Acquisition (PETRA) [1,4,8,12,13].

The PETRA sequence allows 3D isotropic imaging with TEs under 0.1 msec by applying the readout gradients prior to the radiofrequency pulse and combining radial half-projection filling of the outer portions of k-space with single pointwise Cartesian filling of the central portions of k-space, which are otherwise missed during the transmit/receive switching delay [1,7]. PETRA has been applied at 3T for *in vivo* imaging of the knee with a single TE with a focus on meniscus [12,14], cartilage [14], and the cruciate [14] and collateral ligaments [14]. The signal-to-noise ratio (SNR) benefits of UTE and ZTE sequences at 7T compared to lower field strengths have been demonstrated and it has been shown that high SNR can be achieved at high resolution (0.83 mm isotropic) for in vivo human limb/joint imaging at 7T [10].

While other ZTE imaging approaches have been applied in human joints at 7T [10,15], the reported applications of PETRA at 7T in musculoskeletal and other ultrashort-T_2_* tissues have been limited. PETRA has been applied to ex vivo imaging of bovine bone specimens at 7T [4] and is available as a commercial sequence at 7T, but we are unaware of previous literature describing 7T testing and optimization in human joints. Therefore, 7T testing and optimization of the PETRA sequence is needed to evaluate image appearance and potential challenges at this higher field strength.

In addition to minimizing artifacts compared to some other ZTE approaches [4], the Cartesian single point k-space filling approach that PETRA uses allows image acquisition at different k-space gap sizes, corresponding to a range of TEs, as required to perform ultrashort-T_2_* mapping [1,4]. In addition to single-TE PETRA assessment, it would be beneficial to expand the applications of this sequence to ultrashort-T_2_* mapping and to assess the performance of such mapping at 7T. As mentioned above, PETRA-based T_1ρ_ relaxometry [13] has been performed by adding a T_1ρ_ preparation module and performing multiple scans at a series of spin-lock times. T_1ρ_ relaxometry provides information about proteoglycan content [13]. Similarly, ultrashort-T_2_* relaxometry provides quantitative information about tissue degeneration or other changes, potentially related to disruption of normal collagen organization, in ultrashort-T_2_* tissues [16–18]. Ultrashort-T_2_* mapping has been shown to be particularly sensitive to tissue changes and tissue properties in ultrashort-T_2_* tissues such as deep/calcified cartilage [17,18], meniscus [16,19], tendon [20,21], and ligament [22,23]. However, PETRA-based ultrashort-T_2_* mapping has not yet been assessed.

In this work we present ultrashort-T_2_* mapping results using a modified version of a commercially available PETRA sequence which allows an increased TE range of 0.07 to 0.58 msec. Using in vivo knee imaging studies, we performed an initial optimization and addressed the appearance of artifacts at the commercially available sequence settings when using the shortest achievable TE. In addition, we evaluated the feasibility of using the modified extended TE range to perform ultrashort-T_2_* mapping in phantom and in vivo knee images.

## Materials and methods

The source code of the product PETRA sequence was modified by extending the maximum allowable TE from 0.10 msec to 1.10 msec and adjusting the related sequence parameters such as repetition time (TR), size of the cartesian section of the k-space, and sequence timing. The increase in TE values required an increase in the size of the inner k-space portion filled with single pointwise Cartesian approach and a reduction in the outer k-space filled using radial sampling. Together, these changes increased the signal acquisition time, and therefore also the TE. We limited our maximum time for a single scan to 12 minutes for participant tolerance and determined that 0.58 was the maximum TE feasible within this scan time (details provided in the next section). The modified PETRA sequence was implemented on a 7T MAGNETOM Terra (Siemens, Erlangen, Germany) to collect phantom and knee data using a 1-transmit/28-receive-channel knee coil (Quality Electrodynamics, Mayfield Village, Ohio, USA).

### Multi-echo image optimization for ultrashort-T_2_* mapping

The multi-echo image optimization goals included achieving the necessary levels of SNR and artifact reduction to allow ultrashort-T_2_* mapping within clinically feasible scan times, reported as minutes:seconds, at the highest practically achievable spatial resolution within this time limit (S1 Table).

#### In vivo knee imaging.

Eight healthy study participants participated in the study between November 19, 2022 and June 15, 2024. The study was approved by the institutional review board, and written informed consent was obtained from all participants. For the initial optimization studies, five healthy participants (two male, three female, 42 ± 13 years old) were scanned using the modified PETRA sequence for initial optimization. Dielectric pads (7TNS Neuro Set, Multiwave Imaging, Marseille, France) were wrapped around the anterior and posterior surfaces of the knee. All PETRA scans used a single excitation and a flip angle of 6°. In a phantom, a transmit/receive reference amplitude of 175 V allowed the desired 6° flip angle to be achieved. In human participants, the system transmit/receive reference amplitude was adjusted to 170 V, which was experimentally set at the maximum value that avoided exceeding the specific absorption rate limit. Images were subsequently evaluated to confirm that SNR was at a high level. Initial imaging was performed with spatial resolutions ranging from 0.32 to 1.56 mm isotropic and TR values ranging from 3.99 to 8.28 msec. Data with and without chemical shift selective fat saturation [1] were collected to test the effect of fat suppression on data quality. Optimized parameters were selected for 1) a high-resolution series with a single short TE and for 2) a lower-resolution series that made acquisition at extended TE values up to 0.58 msec feasible, given the allowable scan time threshold of 12 minutes. The scan parameters listed in S1 Table were selected as providing the best balance between desired resolution, contrast, and scan time. For all knees, a conventional-TE DESS sequence (TR = 8.68 msec, TE = 2.55 msec, resolution 0.30 mm isotropic, fat suppressed) was also acquired for comparison and to provide a conventional-contrast anatomical reference.

### Ultrashort-T_2_* mapping

#### MnCl_2_ phantom.

A MnCl_2_ phantom was used to perform initial evaluation of the ultrashort-T_2_* mapping protocol using scan parameters based on the initial knee optimization work. The MnCl_2_ phantom contained ten 5 mL centrifuge tubes having 0.02–27.07 mM MnCl_2_ tetrahydrate, based on target ultrashort-T_2_* values estimated using reported relaxivity relationships at 3T [24], and 30 mM NaCl in deionized water [25]. The tubes were arranged within a cylindrical plastic container sized to fit within the knee coil. Plastic supports were used to keep the tubes stationary in the central volume of the container.

Two scan sessions were performed. In the first session, the phantom was imaged with the PETRA sequence using: matrix = 96; FOV = 150mm isotropic; 29,000 radial views; voxel size = 1.56 mm isotropic; flip angle = 6°; receive bandwidth = 365 Hz/pixel; fat suppressed; TR = 7.13 msec; and TE values of 0.07, 0.09, 0.12, 0.16, 0.20, 0.24, 0.30, 0.40, and 0.50 msec. To test within-day repeatability, the TE = 0.07 and 0.50 msec scans were repeated. Acquisition time ranged from 4–5 minutes for TE = 0.07–0.40 msec and was 7 minutes for TE = 0.50 msec. The second session replicated the first session, apart from repeating the TE = 0.07 and 0.50 msec scans.

#### Collagen phantom.

The collagen phantom contained nine 5 mL centrifuge tubes filled with water-collagen solutions having different collagen (chicken hydrolyzed collagen powder, BulkSupplements, Henderson, NV, USA) weight/volume (W/V) percentages (0, 5, 10, 15, 20, 25, 30, 35, and 40%). The tubes were arranged and supported within a cylindrical container in a similar manner to the MnCl_2_ phantom. The container was filled with approximately 1L of 30 mM NaCl in deionized water for coil loading.

Two scan sessions, separated by six weeks, were performed. During both sessions, PETRA series were acquired using the following parameters: total scan time = 5:21; matrix = 160; FOV = 160 mm isotropic; 80,000 radial views; voxel size = 1.00 mm isotropic; flip angle = 6°; bandwidth = 365 Hz/pixel; TR = 4.00 msec; TE = 0.07 msec. During the first scan session, the phantom was imaged with the phantom in two physical orientations. The phantom was rotated about its long axis (which was aligned with the scanner’s z-axis for both scans) by 180° relative to its initial orientation for the second scan. This was done to test for spatial positioning effects on signal homogeneity. During the first scan session, scans with TE values 0.09, 0.12, 0.16, 0.20, 0.24, 0.30, 0.40, and 0.50 msec were also acquired to allow ultrashort-T_2_* mapping. To allow confirmation of which tubes exhibited relatively short versus long T_2_* times, and thus would be reasonable or unreasonable to apply ultrashort-T_2_* mapping to, conventional T_2_* mapping using an axial plane multi-echo GRE sequence was also performed during the first scan session (matrix = 132×134 (264×268 interpolated); FOV = 157×159 mm; pixel size = 0.595×0.593 mm in-plane; slice thickness = 1.20 mm; flip angle = 15°; bandwidth = 600 Hz/pixel; fat suppressed; TR = 37.00 msec; TE = 1.60, 3.81, 5.82, 7.83, 9.84, 11.85, 23.00 msec).

#### In vivo knee imaging.

PETRA imaging, using the parameters listed for the low resolution series in S1 Table and with seven TE values (0.07, 0.09, 0.12, 0.16, 0.22, 0.30, and 0.58 msec; total cumulative scan time for all TE values = 44:03), were performed in three male participants (33 ± 5 years old) with no history of knee injury to test the feasibility of performing ultrashort-T_2_* mapping *in vivo*. Ultrashort- T_2_* mapping repeatability data (two scans separated by 10 days) were acquired for one of these participants.

### Image analysis procedures

#### General.

For all images, 3D Slicer [26] was used to perform segmentation and MATLAB (R2024a, The Mathworks, Natick, MA) was used to measure/calculate results within the desired regions of interest (ROIs).

#### Image segmentation.

ROIs in the knee and image background were manually drawn in 3D Slicer by a researcher with 8 years of knee MRI segmentation experience. For all tissues except cartilage, skin, and cortical bone, the ROIs included the entire tissue of interest in at least one slice where the tissue was clearly visualized. The cartilage segmentation excluded the most medial and lateral slices, where partial volume averaging or blurring occurred. The skin and cortical bone segmentations were limited to central regions within a small set of slices, to avoid thinner tissue regions relative to the scan resolution and the resulting partial volume averaging and the blurring seen in the most medial and lateral slices. For all tissues, regions near the proximal and distal borders of the image were excluded, to avoid signal inhomogeneity near the edges of the coil’s sensitive volume.

For the phantoms, segmentation was performed using the TE = 0.07 msec images. Circular ROIs of 7 mm diameter, centered within each 15 mm diameter centrifuge tube and avoiding regions with air bubbles, were drawn in on six slices within the straight section of the tube.

#### Analysis of Signal Behavior.

Two scans were acquired for one participant to allow for SNR measurement. The SNR was measured in the cortical bone, patellar tendon, posterior cruciate ligament, anterior cruciate ligament, cartilage, and skin in the fat-suppressed low resolution (1.52 mm isotropic) and high resolution (0.34 mm isotropic), TE = 0.07 msec PETRA series. The mean signal in the ROIs described above was calculated. PETRA image noise was measured by calculating the difference between two subsequently acquired identical series, and then calculating the standard deviation of the difference image divided by √2 to correct for the difference operation [27]. Contrast-to-noise ratio (CNR) was also calculated, with the mean signal within a ROI in the adipose tissue posterior to the femur in the most central slice through the posterior cruciate ligament being used as the reference for contrast.

A custom MATLAB script was used to analyze the signal-concentration relationship at TE = 0.07 msec (collagen phantom) and to calculate the ultrashort-T_2_* maps using the TE = 0.07–0.50 msec data (MnCl_2_ phantom and knee images).

#### Ultrashort T_2_* Fitting.

For the MnCl_2_ phantom studies, the ultrashort-T_2_* maps were calculated from 1.50 mm isotropic, fat-suppressed PETRA at nine different TE values (0.07–0.50 msec) using voxel-wise monoexponential fitting in MATLAB. The built-in *lsqcurvefit* function was used, which seeks the best fit based on searching for a minimum in the sum of squares of the residuals with the trust-region-reflective algorithm. Two additional fitting methods, monoexponential fitting with a noise term and log-linear least squares fitting, were also tested for comparison. Monoexponential fitting with a noise term was performed following the same method as the monoexponential fitting. Log-linear least squares fitting was performed by first log transforming the voxel signal intensity at each TE to allow voxel-wise linear least squares non-iterative fitting of the natural log of the signal-intensity versus the array of TEs. The negative of the inverse of the slope of the resulting linear function for each voxel is the estimated ultrashort-T_2_*.

The coefficient of determination, *R*^2^, for the ultrashort-T_2_* fitting was calculated for each voxel. The ROI median and interquartile range of the ultrashort-T_2_* for the MnCl_2_ phantom and signal mean ± standard deviation for the collagen phantom were calculated. The relationships between ultrashort-T_2_* and MnCl_2_ concentration and between signal intensity at TE = 0.07 msec and collagen concentration were evaluated.

For in vivo studies, ultrashort-T_2_* values were calculated using the same process described for the MnCl_2_ phantom, including monoexponential fitting without and with a noise term and log-linear least squares fitting, except that the 1.52 mm isotropic, fat-suppressed PETRA at seven TE values (0.07–0.58 msec) were used (due to the need for a shorter total scan time for in vivo imaging). In addition, the images were carefully visually inspected using semi-transparent image overlay visualization to confirm that there was no participant movement between acquisition of the image volumes with different TE values. In addition to the ultrashort-T_2_* fitting performed with all seven acquired image volumes, ultrashort-T2* fitting was also performed with a subset of three TE values (0.07, 0.12, and 0.30 msec). The goal of using only 3 TE values was to reduce the total scan time (16:43), thereby minimizing participant motion between series and increasing the practical feasibility. In addition, in-plane frequency-domain interpolation was performed in MATLAB prior to segmentation and ultrashort-T_2_* fitting to allow more precise delineation of the knee structures (resulting in a sagittal-plane series interpolated resolution of 0.76 x 0.76 x 1.52 mm). Median ultrashort-T_2_* values within each ROI were calculated once using all ROI voxels, and again after voxels for which *R*^*2*^ was < 0.5 were excluded. The ultrashort-T_2_* values and *R*^*2*^ values were measured in the cortical bone, patellar tendon, meniscus, posterior cruciate ligament, anterior cruciate ligament, cartilage, and skin. *X*^2^ was also calculated to allow more robust comparison between goodness-of-fit between the fitting using seven TE values and three TE values.

## Results

### Multi-echo image optimization for ultrashort-T_2_* mapping

#### In vivo knee imaging.

**Fig 1:**
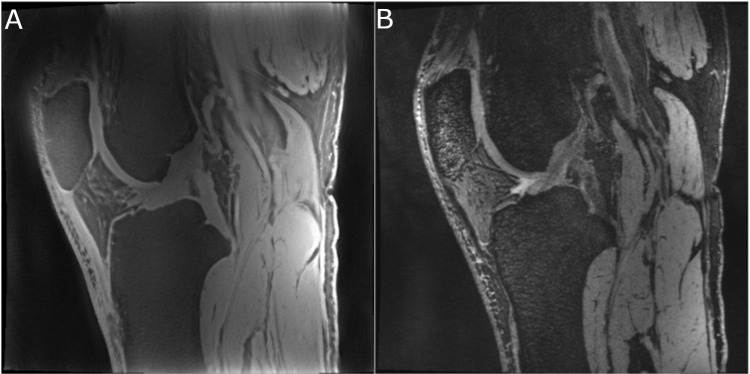
Example high-resolution PETRA knee image, with comparison to a conventional-TE DESS sequence image. Sagittal images of one participant’s knee from the fat-suppressed PETRA series at 0.34 mm isotropic resolution (A) and from the fat-suppressed dual-echo steady state (DESS) at 0.30 mm isotropic resolution (B). These images illustrate the difference in signal, and resulting differences in contrast between conventional and zero echo-time sequences, in the patellar tendon, cruciate ligaments, and other ultrashort-T_2_* tissues in these images. Zero echo-time series can enhance visualization of certain structures, supplementing the visualization provided by series with more conventional tissue contrast.

Fig 1 shows an example high-resolution PETRA knee image, with comparison to a dual-echo steady state (DESS) conventional TE sequence image for anatomical reference with more standard tissue contrast. The PETRA images were obtained with high SNR, even for ultrashort-T_2_* tissues. As listed in Table 1, the SNR in the cortical bone, patellar tendon, meniscus, posterior cruciate ligament, anterior cruciate ligament, cartilage, and skin in the TE = 0.07 msec, 1.52 mm isotropic resolution, TR = 4.21 msec series ranged from 68 to 337 (total scan time, 5:20). In the 0.34 isotropic resolution, TR = 7.07 msec series (Fig 1), the same SNR measurements ranged from 23 to 75 (total scan time, 6:55). Note that the PETRA series at the two resolutions were acquired with different TR, so the SNR does not follow the usual relationship with voxel size between the two series, and CNR was also impacted. The 1.52 mm resolution series had higher SNR efficiency (SNR per unit time) and CNR. For comparison to a more standard-contrast image series, the SNRs in these regions for the 0.30 mm isotropic resolution conventional-TE DESS series ranged from 16 to 30.

**Table 1:** SNR, SNR efficiency, and CNR measurements for PETRA at TE = 0.07 msec for knee tissue ROIs at two resolutions.

	Results TR = 7.07 msec and 0.34 mm isotropic resolution			Results for TR = 4.21 msec and 1.52 mm isotropic resolution		
Knee tissue	SNR	SNR efficiency (SNR per minutes of scan time)	CNR	SNR	SNR efficiency (SNR per minutes of scan time)	CNR
Cortical bone	23	3.3	2	68	12.8	6
Patellar tendon	58	8.4	33	183	34.3	108
Meniscus	36	5.2	11	139	26.1	64
Posterior cruciate ligament	39	5.6	14	163	30.6	88
Anterior cruciate ligament	34	4.9	9	143	26.8	69
Cartilage	52	7.5	27	172	32.3	98
Skin	75	10.8	50	337	63.2	263

**Fig 2:**
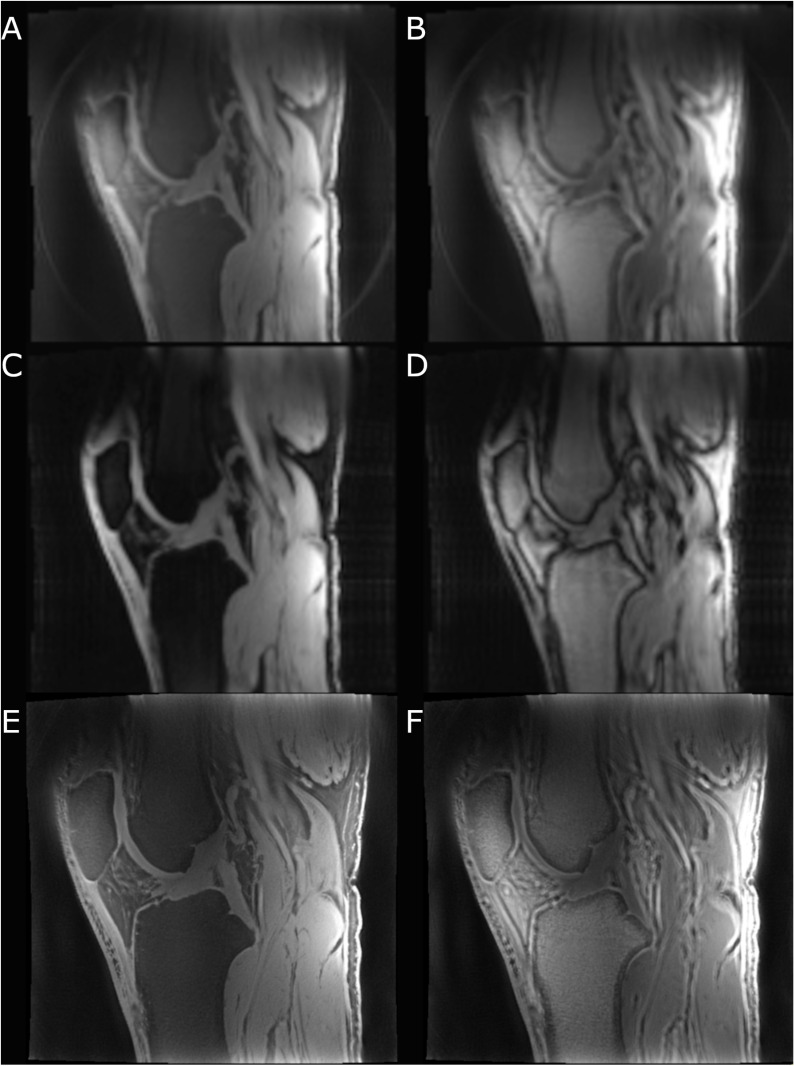
Sagittal PETRA images all acquired in a single participant knee, acquired using the parameters listed in S1 Table. Low-resolution images acquired at TE = 0.07 msec with (A) and without (B) fat suppression, at TE = 0.58 msec with (C) and without (D) fat suppression, and high-resolution images at TE = 0.07 msec with (E) and without (F) fat suppression. Note the off-resonance artifacts appearing as double lines and thick low/high signal borders around tissue interfaces (e.g., patellar tendon-infrapatellar fat pad interface, muscle-fat interfaces, etc.).

Comparing the images acquired with and without fat suppression revealed that fat suppression reduced the appearance of off-resonance artifacts and improved the visualization of short-T_2_* tissues (Fig 2). Images with 0.34 mm isotropic resolution were acquired in scan times of 6:55 when acquired with fat suppression and 6:00 when acquired without fat suppression. Fig 2 shows example low-resolution images acquired at TE = 0.07 and 0.50 msec and high-resolution images acquired at TE = 0.07 msec, with and without fat suppression.

To achieve acquisitions at longer TE values without exceeding 12 minutes per scan, the resolution needed to be set to 1.52 mm isotropic. At this resolution with 70,000 radial views and TR = 4.21 msec the scan time was under 12 minutes at TE values up to 0.58 msec (scan time = 10:55, with fat suppression).

### Ultrashort-T_2_* mapping

#### MnCl_2_ phantom.

**Fig 3:**
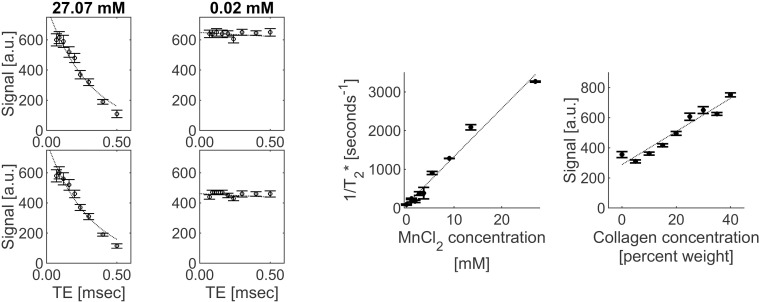
MnCl_2_ phantom plots. Plots showing examples of median measured (round markers) and fitted signal (dashed line) based on estimated ultrashort-T_2_* signal versus TE for three MnCl_2_ phantom solutions from two (top and bottom plots for each region) scans (a), the relationship between mean 1/T2* and MnCl2 concentration, averaged over two scans (b), the relationship between mean signal and collagen concentration at TE = 0.07 msec, averaged over three scans (c). Error bars represent the interquartile range within each subregion for signal versus TE plots and the standard deviation between scans for the 1/T_2_* and signal versus concentration plots.

Monoexponential fitting without a noise term generally produced more homogeneous ultrashort-T_2_* within each ROI and smaller inter-scan differences than the other two fitting methods, and so are presented as the main results. The monoexponential-fit without noise term results for ultrashort-T_2_* measurements, ultrashort-T_2_* curve fit *R*^*2*^ values, and absolute and percent change between scans for each phantom solution ROI for two sessions are shown in Table 2. Bland-Altman analysis results are shown in S10 Fig and S11 Table. The same results for the other two fitting methods are shown in S2 Table and S3 Table. Example ultrashort-T_2_* curve fits are shown in Fig 3A. There was a significant linear correlation between 1/T_2_*_ultrashort_ values and MnCl_2_ concentration (*r* = 0.99 and *p* ≤ 0.01 for both scans), as shown in Fig 3B. Mean *R*^*2*^ dropped sharply for MnCl_2_ concentrations below 3.6 mM, and at the lowest concentration (0.02 mM) a large number of very high or even negative ultrashort-T_2_* values was calculated due to the lack of signal decay measured over the range of acquired TEs, leading to poor fit quality and the very large interquartile range for that measurement. The scan 1 and scan 2 slopes and intercepts were calculated using just the results for the solution concentrations with average *R*^*2*^ for ultrashort-T_2_* fit ≥ 0.85 (which corresponded to 5.4–27.07 mM, with ultrashort-T_2_* values of ≤ 1.14 msec). The scan 1 and scan 2 slopes were 108.68 and 109.41 seconds^-1^/mM (<1% difference between scans), and the intercepts were 413.97 and 359.81 seconds^-1^ (13% difference between scans).

**Table 2:** Calculated ultrashort-T_2_* values, between-scan absolute and percent change, and ultrashort-T_2_* fit *R*^2^ from two scans for the MnCl_2_ phantom. The median and interquartile range values are calculated for the sample of all voxels within each ROI.

Phantom MnCl_2_ solution concentration [mM]	Scan 1 T_2_* median (interquartile range) [msec]	Scan 2 T_2_* median (interquartile range) [msec]	Between- scan T_2_* absolute change [msec]	Between- scan T_2_* percent change	Scan 1 mean *R*^*2*^	Scan 2 mean *R*^*2*^
27.07	0.31 (0.03)	0.31 (0.03)	0.00	1%	0.96	0.96
13.53	0.47 (0.04)	0.49 (0.05)	0.02	5%	0.98	0.95
9.01	0.78 (0.10)	0.78 (0.06)	0.00	0%	0.86	0.88
5.4	1.07 (0.20)	1.14 (0.20)	0.07	7%	0.97	0.95
3.6	3.55 (3.90)	2.03 (0.61)	1.52	43%	0.50	0.84
2.7	2.91 (2.87)	2.45 (0.68)	0.47	16%	0.54	0.73
1.79	5.12 (2.17)	6.61 (1.80)	1.49	29%	0.34	0.24
1.12	4.73 (0.73)	6.08 (2.19)	1.34	28%	0.37	0.30
1.07	3.96 (2.23)	4.32 (1.87)	0.36	9%	0.56	0.64
0.02	12.34 (9.61)	9.89 (10.40)	2.44	20%	0.08	0.11

#### Collagen phantom.

For the collagen phantom, the mean conventional-T_2_* values for all W/V percentages greatly exceeded the ultrashort-T_2_* upper limit, being 23 msec for 40% collagen to 330 msec for 5% collagen; therefore ultrashort-T_2_* mapping was not evaluated. There was a significant linear correlation between collagen concentration and signal intensity in the TE = 0.07 msec images (*r* = 0.98 on average, *p* <0.0001). The individual signal measurements, linear fitting parameters, and correlation statistics are shown in Table 3. Fig 3C shows this relationship for the average of the three TE = 0.07 msec PETRA series. The between-scan and between-orientation signal and linear fitting parameter CV were all ≥ 0.05 except for 0% collagen signal (Table 3). Bland-Altman analysis results are shown in S9 Fig and S11 Table.

**Table 3:** Signal measurements for each collagen concentration from three scans of the collagen phantom and the resulting signal versus concentration linear fitting parameters and correlation statistics.

	Scan 1, orientation 1	Scan 1, orientation 2	Scan 2, orientation 1	CV
Collagen W/V percentage [%]	Mean signal (standard deviation) measurements [a.u.]			
0	301 (32)	268 (16)	355 (20)	14%
5	307 (16)	309 (15)	310 (10)	1%
10	389 (13)	403 (23)	362 (10)	5%
15	463 (11)	455 (25)	417 (10)	5%
20	520 (21)	481 (25)	496 (13)	4%
25	604 (19)	564 (19)	607 (24)	4%
30	637 (16)	627 (19)	651 (23)	2%
35	680 (21)	646 (14)	625 (9)	4%
40	792 (30)	719 (43)	752 (13)	5%
Linear fit and correlation parameter values				
Slope [a.u./%]	12.40	11.26	11.01	6%
Intercept [a.u.]	273.34	271.74	288.32	3%
*r*	0.99	0.99	0.96	
*P*	<0.0001	<0.0001	<0.0001	

#### In vivo knee imaging.

**Fig 4:**
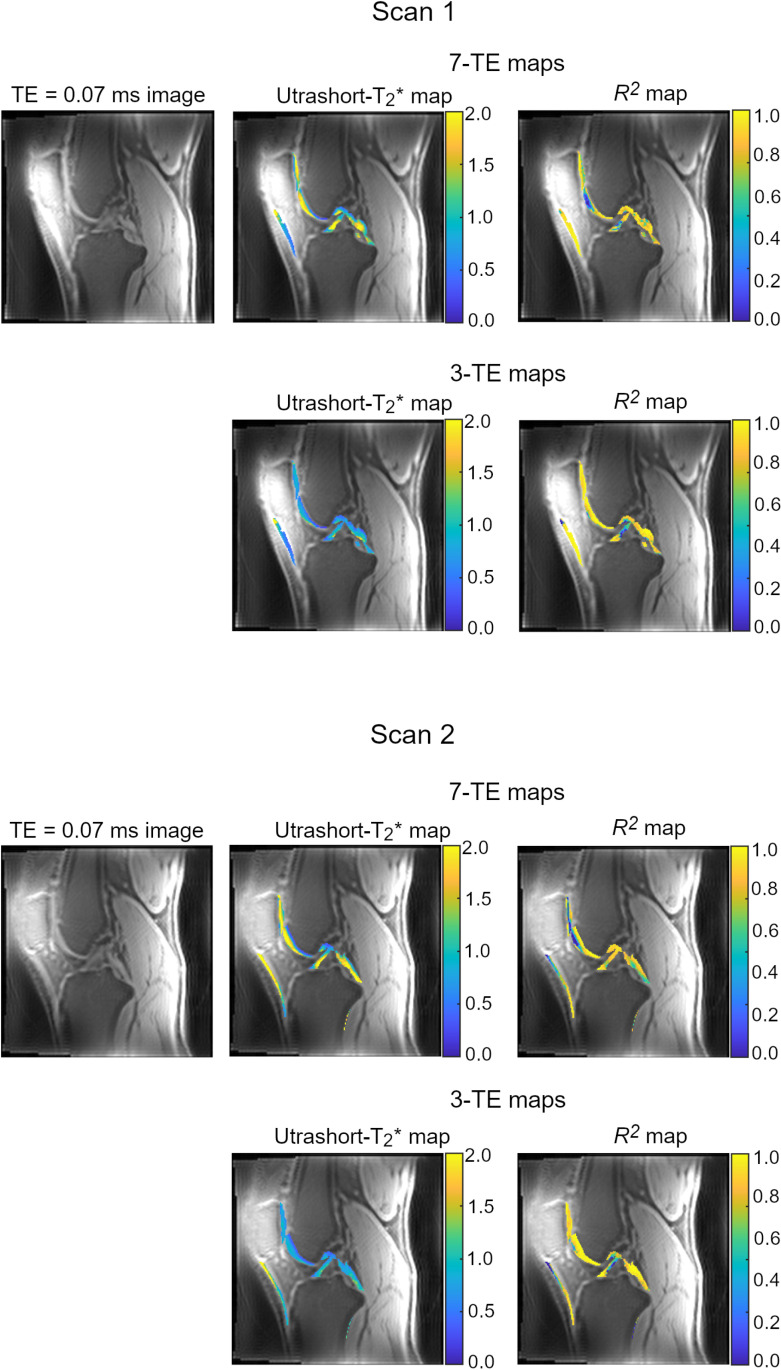
Ultrashort-T_2_* map and corresponding *R*^2^ map for one knee for two different scan sessions. Images and maps for one participant’s knee scanned in two sessions, grouped by scan session and labeled as Scan 1 (top two rows) and Scan 2 (bottom two rows). Images for each scan include an example PETRA image at TE = 0.07 msec, ultrashort-T_2_* map in msec calculated using 7 TE values and *R*^2^ fit goodness map for the 7-TE fit in the masked ROIs (upper row of each set), and the ultrashort-T_2_* map in msec calculated using 3 TE values and *R*^2^ fit goodness map for the 3-TE fit in the masked ROIs (bottom row of each set).

**Fig 5:**
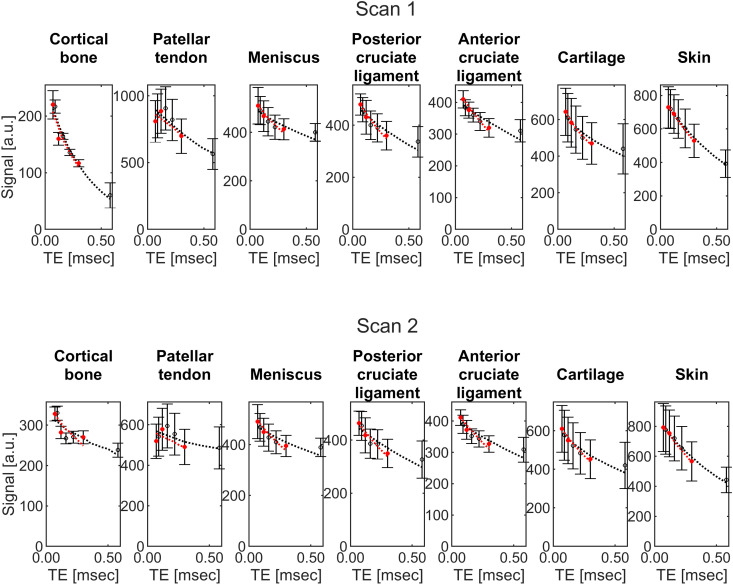
Example T_2_* curves. Plots of the median measured (round markers) and fitted (dashed line) signal based on estimated ultrashort-T_2_* signal versus TE for the measured knee tissue regions from two scans of a single participant’s knee (10 days between scans). The points and for signal decay curves fitted to all seven TE values are shown in black and the points and signal decay curves fitted to a subset of three TE values are shown in red. Error bars represent the interquartile range within each subregion.

Acquiring images with different TEs but other parameters held constant allowed creation of ultrashort-T_2_* maps. Fig 4 shows an ultrashort-T_2_* map and corresponding *R*^*2*^ map for one knee for two different scan sessions. Ultrashort-T_2_* measurements and fitting information for the cortical bone, patellar tendon, meniscus, posterior cruciate ligament, anterior cruciate ligament, cartilage, and skin are shown in Table 4. The percent of voxels excluded due to *R*^2^ being below 0.5 are listed for each tissue in Table 5. Scan-rescan results for one participant and interscan coefficients of variation are shown in Table 6 and Bland-Altman analysis results are shown in S10 Fig and S11 Table. Results obtained using all seven acquired TE values and using a subset of three TE values are presented for both tables. Example T_2_* curves are shown in Fig 5. The same sets of results obtained using monoexponential fitting with the addition of a noise term and log-linear least squares fitting are presented in S4 Table, S5 Table, S6 Table, and S7 Table.

**Table 4:** Knee tissue calculated median and range ultrashort-T_2_* values, ultrashort-T_2_* fit *R*^2^, and ultrashort-T_2_* fit *X*^2^ for single scans for three participants. The results calculated using all seven TE values and the subset of three TE values are presented. The median value represents the median of the three median values (one per participant, for each ROI) calculated for each ROI. The range represents the minimum and maximum participant ROI median values.

Knee tissue	Median ultrashort-T_2_* (Range) [msec]	Median *R*^2^ (Range)	Median *X*^2^ (Range)	Median ultrashort-T_2_* (Range) [msec]	Median *R*^2^ (Range)	Median *X*^2^ (Range)
Results when fitting to seven TE values						
	All voxels			Voxels with *R*^2^ ≥0.5		
Cortical bone	0.7 (0.35–1.34)	0.93 (0.71–0.93)	6.36 (3.71–9.38)	0.7 (0.4–1.29)	0.93 (0.82–0.93)	4.26 (3.71–9.38)
Patellar tendon	1.17 (1.06–1.57)	0.90 (0.71–0.93)	5.75 (3.48–18.45)	1.07 (1.01–1.29)	0.92 (0.81–0.94)	5.15 (3.16–15.93)
Meniscus	1.95 (1.75–2.29)	0.59 (0.58–0.62)	8.39 (8.3–8.95)	1.77 (1.57–2.11)	0.77 (0.73–0.78)	6.12 (5.92–6.51)
Posterior cruciate ligament	1.38 (1.25–2.27)	0.79 (0.76–0.81)	8.79 (3.54–10.37)	1.31 (1.27–2.25)	0.82 (0.81–0.84)	8.21 (2.86–8.41)
Anterior cruciate ligament	1.5 (0.97–1.94)	0.71 (0.35–0.84)	9.89 (7.85–13.33)	1.3 (0.76–1.66)	0.81 (0.77–0.9)	5.77 (4.91–10.81)
Cartilage	1.05 (0.9–1.22)	0.87 (0.75–0.9)	10.54 (7.39–19.2)	0.97 (0.8–1.09)	0.91 (0.82–0.93)	7.12 (6.81–14.76)
Skin	0.82 (0.76–0.98)	0.98 (0.97–0.98)	3.73 (2.27–4.55)	0.82 (0.76–0.96)	0.98 (0.97–0.98)	3.63 (2.27–4.29)
Results when fitting to three TE values						
	All voxels			Voxels with *R*^2^ ≥0.5		
Cortical bone	0.75 (0.35–0.75)	0.87 (0.83–0.89)	1.2 (1.05–3.61)	0.75 (0.35–0.75)	0.87 (0.87–0.89)	1.05 (0.86–3.61)
Patellar tendon	1.01 (0.94–1.58)	0.89 (0.52–0.93)	1.03 (0.63–5.24)	0.80 (0.80–0.96)	0.97 (0.87–0.98)	0.48 (0.35–2.98)
Meniscus	0.91 (0.89–1.03)	0.82 (0.76–0.88)	1.98 (1.33–2.18)	0.89 (0.80–1.00)	0.88 (0.8–0.89)	1.76 (1.22–1.80)
Posterior cruciate ligament	0.76 (0.73–1.13)	0.91 (0.49–0.93)	1.59 (1.36–2.16)	0.75 (0.72–1.00)	0.92 (0.79–0.96)	1.4 (0.71–1.54)
Anterior cruciate ligament	0.79 (0.60–0.84)	0.89 (0.56–0.98)	1.62 (0.66–3.98)	0.75 (0.53–0.82)	0.92 (0.91–0.99)	0.89 (0.29–1.23)
Cartilage	0.71 (0.67–0.72)	0.95 (0.91–0.95)	0.97 (0.94–2.34)	0.68 (0.66–0.70)	0.96 (0.93–0.96)	0.85 (0.81–1.95)
Skin	0.74 (0.73–0.82)	0.98 (0.98–0.99)	0.42 (0.25–0.43)	0.74 (0.72–0.82)	0.98 (0.98–0.99)	0.42 (0.25–0.43)

**Table 5:** Percent voxels excluded for *R*^2^ being below the 0.5 threshold for single scans for three participants. The results for fitting using all seven TE values and the subset of three TE values are presented.

Knee tissue	Median (range) of percent voxels excluded for *R*^*2*^ < 0.5 [%]	
	Results when fitting to seven TE values	Results when fitting to three TE values
Cortical bone	0 (0–30)	0 (0–17)
Patellar tendon	13 (7–25)	31 (26–49)
Meniscus	39 (37–42)	14 (9–27)
Posterior cruciate ligament	18 (6–24)	17 (3–51)
Anterior cruciate ligament	30 (29–60)	21 (16–47)
Cartilage	24 (10–25)	7 (6–11)
Skin	2 (0–3)	0 (0–1)

**Table 6:** Scan 1 and scan 2 single-participant ROI median and interquartile range (IQR) ultrashort-T_2_* values and between-scan absolute and percent change calculated from the two scan sessions for one participant. The results calculated using all seven TE values and the subset of three TE values are presented for all voxels and for only voxels with acceptable ultrashort-T_2_* fit (R^2^ ≥ 0.5). The median and interquartile range values are calculated for the sample of all voxels within each ROI.

	Scan 1 median ultrashort-T_2_* (IQR) within each ROI [msec]		Scan 2 median ultrashort-T_2_* (IQR) within each ROI [msec]		Scan-rescan ultrashort-T_2_* absolute change [ms]		Scan-rescan ultrashort-T_2_* percent change	
Knee tissue	All voxels	Voxels with *R*^2^ ≥0.5	All voxels	Voxels with *R*^2^ ≥0.5	All voxels	Voxels with *R*^2^ ≥0.5	All voxels	Voxels with *R*^2^ ≥0.5
Results when fitting to seven TE values								
Cortical bone	0.35 (0.05)	0.35 (0.05)	1.77 (1.29)	1.69 (1.22)	1.44	1.34	398%	378%
Patellar tendon	1.57 (1.41)	1.29 (0.84)	4.43 (1996.69)	1.46 (1.10)	2.85	0.17	182%	13%
Meniscus	1.75 (1.32)	1.57 (1.03)	1.95 (1.37)	1.73 (0.85)	0.20	0.16	12%	10%
Posterior cruciate ligament	1.25 (0.75)	1.27 (0.77)	1.30 (0.78)	1.26 (0.78)	0.04	0.01	3%	1%
Anterior cruciate ligament	1.50 (1.06)	1.30 (0.87)	1.56 (0.92)	1.35 (0.72)	0.06	0.05	4%	4%
Cartilage	1.22 (1.16)	1.09 (1.08)	1.40 (1.04)	1.18 (0.94)	0.18	0.09	15%	9%
Skin	0.76 (0.33)	0.76 (0.33)	0.92 (0.35)	0.92 (0.35)	0.16	0.16	21%	20%
Results when fitting to three TE values								
Cortical bone	0.35 (0.09)	0.35 (0.09)	0.97 (0.41)	0.74 (0.38)	0.62	0.38	174%	108%
Patellar tendon	1.58 (2.57)	0.96 (0.53)	3.03 (8.62)	1.08 (0.45)	1.46	0.12	92%	13%
Meniscus	0.89 (0.48)	0.80 (0.34)	0.94 (0.38)	0.86 (0.26)	0.05	0.06	5%	8%
Posterior cruciate ligament	0.73 (0.32)	0.72 (0.31)	0.75 (0.27)	0.74 (0.26)	0.02	0.02	3%	3%
Anterior cruciate ligament	0.79 (0.29)	0.75 (0.27)	0.82 (0.25)	0.79 (0.24)	0.03	0.04	4%	5%
Cartilage	0.72 (0.46)	0.68 (0.44)	0.77 (0.34)	0.74 (0.32)	0.05	0.06	7%	8%
Skin	0.73 (0.22)	0.72 (0.22)	0.74 (0.20)	0.74 (0.20)	0.02	0.02	3%	3%

## Discussion

We modified a commercially available ZTE sequence, PETRA [1], to extend the maximum technically allowable echo time to up to 1.10 msec and demonstrate the feasibility of extended TE imaging (up to 0.58 msec) with PETRA at 7T in MnCl_2_ and collagen phantoms and in vivo. PETRA imaging was feasible in vivo within clinically realistic acquisition times at high resolution (0.34 mm isotropic) at short TE values and lower resolution (1.52 mm isotropic) at the high TE values. In addition to distinguishing between tissues and tissue components with comparatively short T_2_* values from those with long T_2_* values, these modifications further allow advanced applications such as ultrashort-T_2_* mapping from 7T ZTE data.

### Image quality indicators

Higher SNR was measured in all acquired PETRA images than in a conventional TE series at similar resolution. Fat suppression was useful for reducing off-resonance artifact. As previously described [10], coil artifact was observed in a few instances. However, it was outside of the knee volume and therefore did not impact image quality. Furthermore, near-isocenter positioning of the knee allows for minimization of lateral image blurring, as reported previously [28].

### Ultrashort T_2_* mapping

The extension of the maximum TE allows ultrashort-T_2_* mapping by multiple acquisitions for substances with sub-millisecond T_2_* values, demonstrated here using the MnCl_2_ phantom and in vivo knees. Ultrashort-T_2_* fitting *R*^*2*^ was ≥0.86 for MnCl_2_ phantom solutions with ultrashort-T_2_* ≤ 1.14 msec and for most of the knee tissues analyzed. For MnCl_2_ samples, there was a strong significant correlation between 1/ultrashort-T_2_* values and concentration. Although this finding is not surprising, given the predictions of fast exchange theory, the linearity of the relaxivity plot provides an important quality verification of the T_2_* mapping capabilities of the sequence.

We simulated collagen-rich tissues using a collagen phantom. Although the collagen concentrations were similar to the range found in typical short-T_2_* tissues, the lack of collagen organization limits the phantom’s similarity to actual tissue and resulted in measured conventional T_2_* values that were much longer than in the tissues of interest [29]; therefore, ultrashort-T_2_* mapping was not evaluated. However, we observed a strong correlation between collagen concentration and signal at TE = 0.07 msec, likely due to T_1_ differences between the collagen solutions. Previously reported collagen solution T_1_ values suggest that the expected T_1_ range for our 5%-40% collagen solutions would be around 1.6–2.6 sec at 3T [30] and somewhat longer at 7T [31–33]. The T_1_ values in short-T_2_* musculoskeletal and skin tissues with would be expected to be around 330–350 (bound water)/390–1000 (pore water) msec in cortical bone at 4.7 T [34,35], 620–700 msec in tendon at 3T [3,36,37], 819–870 msec in ligament at 3T [37], 870 msec in meniscus at 3T [37], and 1.13 sec in cartilage at 3T [37], and 820–1060 msec in dermis and epidermis of the skin at 1.5T [38], with relatively longer T_1_ values expected for each tissue at 7T. This range of T_1_ values would also be expected to create collagen-dependent signal variations in similarly configured PETRA images. While variations in other structural and compositional tissue properties would prevent across-tissue interpretation of the signal variations as reflecting collagen content, it is possible that within-tissue signal variations could be interpretable as variations in collagen content and could be associated with tissue pathology. Future work with relevant tissues is needed to determine if this application is possible, as the collagen solutions used in this work do not reflect the structure or complexity of actual tissue.

The median ultrashort-T_2_* values were generally similar between the three participants. When comparing ultrashort-T_2_* values calculated using all seven versus the subset of three TE values we found that the median ultrashort-T_2_* values were lower when only three TE values were used than when seven were used (maximum TE = 0.30 msec versus 0.58 msec). This may reflect higher contributions of shorter T_2_* components when shorter TE values are used, with longer components contributing more to the overall measurement as a greater proportion of longer TE values are included. Future work in tissue samples or patients is needed to determine if the differences in ultrashort-T_2_* values calculated using 7 versus 3 TE values are meaningful.

The use of three TE values for ultrashort-T_2_* fitting resulted in better fit and reproducibility than when seven TE values were used. Of the tested tissues, cortical bone showed the poorest reproducibility, which may be in part due to the thin ROI relative to the low scan resolution and potential for chemical shift artifact around the bone, and the relatively distal location of the bone ROI relative to the image volume center. Midshaft bone measurements, where the cortical bone is thicker and the bone ROI can be centered in the imaging volume, may be more reproducible. The shorter scan time, better decay curve fit, and higher between-scan reproducibility makes the three TE approach preferable for mapping of the ultrashort-T_2_* components. Regardless of the number of TE values used, the maximum TE of ≤0.58 msec used in this work prevents the capture and analysis of long- T_2_* components. The maximum TE value is limited in this sequence, as image quality was markedly reduced when TE values above 0.58 msec were tested. The addition of a conventional-TE T_2_* sequence would be necessary to capture these long-T_2_* components, when desired.

For patellar tendon, the median value of 1.07 msec when seven TE were used and 0.80 msec when 3 TE values were used were lower than the value of 1.57 ± 0.11 msec reported for the short T_2_* component in one patellar tendon sample at 7T [39]. Our values calculated using three TE were of similar magnitude to those reported for short-component T_2_* in the Achilles tendon of healthy volunteers at 7T, which ranged from 0.20–0.48 msec [20]. Tendon long axis angle relative to the main magnetic field was not reported in that work, limiting comparison to the patellar tendon due to the different anatomical orientation of the tendons. We did see some non-exponential behavior in the signal decay of the tendon ROI in some cases (as illustrated in Fig 5), which may be due to partial volume averaging with adjacent adipose tissue or blurring where the tendon is too far from isocenter.

The monoexponential T_2_* of cartilage in uninjured and ACL-reconstruction knees, measured with a mix of ultrashort and conventional TE (TE range: 0.032–16 msec) at 3T, has been reported to be around 14–20 msec [40], and the short/long-T_2_* components of human cadaveric patellar cartilage by biexponential fitting (TE range: 0.08–40 msec) at 3T and 1^st^/2^nd^/3^rd^ components of bovine nasal cartilage by triexponential fitting (TE range: 0.6–614.4 msec) at 9.4T have been reported to be about 0.50/35 msec and 2.3/25.2/96.3 msec respectively [41]. Our cartilage ultrashort-T_2_* values of 0.97 msec (seven TE) and 0.68 msec (three TE) were measured with a sub-millisecond maximum TE, causing a strong ultrashort component weighting, and as a result are more similar to the reported short-component biexponential and triexponential T_2_*values than the longer monoexponential T_2_* values. Ultrashort-T_2_* components may be more sensitive to some pathology than the long components [22].

Conventional- and ultrashort-T_2_* mapping has been less thoroughly explored in skin than it has been in musculoskeletal tissues, but it is of interest since skin is a high-collagen-content tissue that may be impacted alongside musculoskeletal tissues when collagen structure or function is abnormal. Skin conventional-T_2_*, calculated from images acquired at TE values of 2.8–60 msec, has been reported to be 9.8 msec at 1.5T [42,43]. Our measured value of 0.82 msec (seven TE)/0.74 msec (three TE) likely reflects only the shorter components since we only acquired images at sub-millisecond TE values.

Our cortical bone ultrashort-T_2_* median value of 0.70 msec (seven TE)/0.75 msec (3 TE) was similar to the previously reported bone specimen ultrashort-T_2_* values of 0.4–0.7 msec measured at 7T (TE range: 0.064–2.048 msec) [6] and near the bound-water ultrashort-T_2_* values measured previously at 4.7T [35].

The meniscus ultrashort-T_2_* mapping results for both sets of TE (three and seven TE) are both lower than ultrashort-T_2_* at 3T (7.3 msec) [19] and short-T_2_* values at 7T (7.31–9.19 msec) [44] reported previously for healthy menisci. However, our values were within the range of 0.4–4 msec reported previously for short-component T_2_* of meniscus *in vitro* at 9.4T [45]. Similarly, our ACL and PCL ultrashort-T_2_* values were lower than the previously reported values [46]. This is likely in part due to the expected reduction in T_2_* at 7T compared to 3T [47]. As noted above, the extent of the field strength-dependence has been shown to vary by region within tendon [20], and this may also be true in the two cruciate ligaments.

The differences between the results here for all tissues and those reported in the literature are also likely due to the difference in TE range used, where most other studies have been performed with either a single or a few ultrashort-TE and a larger number of conventional TE values (>1 msec), in contrast to our use of only ultrashort-TE values. In addition, our use of a coefficient of determination threshold for excluding poor-fit voxels is less commonly used than approaches in which voxels are excluded for having T_2_* or T_2_ values outside of an expected measurement range. While the use of bicomponent exponential fitting and models with incorporation of Gaussian decay and oscillating off-resonance components have been found to improve the fit of tendon [48,49] and meniscus [45] T_2_* signal decay, these more complex models were not explored in this current work, as the upper range of TE values and number of TE points acquired were not sufficient to enable this more complex modeling. However, these components do likely contribute to the signal behavior and signal change between TE values. These factors, along with partial volume averaging and potential issues due to movement between series, artifact, or noise, may contribute to the poor monoexponential fit in some voxels.

## Limitations

This work was limited to imaging of MnCl_2_ and collagen phantoms and a small number of healthy volunteers. Additional image optimization and parameter adjustment may be needed for optimized imaging of patients, which could be performed based on quantitative measures such as SNR and CNR and reader-based image quality and structure/pathology visualization assessments as described in previous work performed by others [50–52]. The sensitivity and specificity of PETRA-based short-TE signal measurements and ultrashort-T_2_* mapping for quantifying tissue health remains to be assessed. The small sample size and the strong impact on ultrashort-T_2_* of the TE values used here limits the direct comparison between the reported values and our measurements.

PETRA’s combination of pointwise and radial sampling varies depending on TE, and this is expected to have some impact on the measured signal. Future work, using a phantom with known ultrashort-T_2_* values or with tissue samples or participants being imaged with both PETRA and a reference ultrashort-T_2_* measurement sequence, such as FID-based ultrashort-T_2_* calculations [34], may be needed to assess the impact on the accuracy of PETRA ultrashort-T_2_* measurements.

Ultrashort-T_2_* mapping using PETRA does require relatively long cumulative scan time and low resolution to avoid unrealistic scan times. We found that the maximum TE needed to be restricted to 0.58 msec to avoid extremely high scan times for single scans. Bicomponent or multicomponent T_2_* mapping for musculoskeletal tissues would require a wider range of TE values, since long-T_2_* tissue suppression by subtraction imaging and quantitative ultrashort-T_2_* mapping require at least one TE long enough, generally >1 msec, for signal of the short-T_2_* tissue of interest to significantly decrease compared to the long-T_2_* tissue values [3,20,41]. A potential solution to the cumulative scan time with long TE values may be to fix the central portions of k-space filled by the single pointwise approach to an upper limit as function of TE. In this manner, scan time would be shortened while maintaining image resolution and SNR. Other methods, such as iterative reconstruction or deep learning algorithms could also be used for improved efficiency [53–55].

## Conclusion

In this work we present the results of phantom and in vivo knee imaging using a modified PETRA sequence with an increased TE range. The results obtained are encouraging, demonstrating that following suitable image optimization, this updated PETRA sequence can provide high quality in vivo images. Furthermore, ultrashort-T_2_* mapping was feasible using the modified extended TE range in the phantoms and in vivo in a small sample of healthy knees. Work is in progress to reduce scan time at long TEs by setting the Cartesian central region of the k-space to an upper limit while preserving image information.

## Supporting information

S1 Table PETRA scan parameters selected as optimal for visualizing knee structures within a reasonable scan time. (DOCX)

S2 Table Results based on monoexponential fitting with a noise term for ultrashort-T_2_* values, between-scan absolute and percent change, and ultrashort-T_2_* fit *R*^2^ from two scans for the MnCl_2_ phantom. The median and interquartile range values are calculated for the sample of all voxels within each ROI.
(DOCX)

S3 Table Results based on log-linear least squares fitting for ultrashort-T_2_* values, between-scan absolute and percent change, and ultrashort-T_2_* fit *R*^2^ from two scans for the MnCl_2_ phantom. The median and interquartile range values are calculated for the sample of all voxels within each ROI.
(DOCX)

S4 Table Results based on monoexponential fitting with a noise term for knee tissue calculated median and range ultrashort-T_2_* values, ultrashort-T_2_* fit *R^2^*, and ultrashort-T_2_* fit *X^2^* for single scans for three participants. The results calculated using all seven TE values and the subset of three TE values are presented. The median value represents the median of the three median values (one per participant, for each ROI) calculated for each ROI. The range represents the minimum and maximum participant ROI median values.
(DOCX)

S5 Table Results based on log-linear least squares fitting for knee tissue calculated median and range ultrashort-T_2_* values, ultrashort-T_2_* fit *R^2^*, and ultrashort-T_2_* fit *X^2^* for single scans for three participants. The results calculated using all seven TE values and the subset of three TE values are presented. The median value represents the median of the three median values (one per participant, for each ROI) calculated for each ROI. The range represents the minimum and maximum participant ROI median values.
(DOCX)

S6 Table Results based on monoexponential fitting with a noise term for scan 1 and scan 2 single-participant ROI median and interquartile range (IQR) ultrashort-T_2_* values and between-scan absolute and percent change calculated from the two scan sessions for one participant. The results calculated using all seven TE values and the subset of three TE values are presented for all voxels and for only voxels with acceptable ultrashort-T_2_* fit (*R^2^* ≥ 0.5). The median value represents the median of the three median values (one per participant, for each ROI) calculated for each ROI. The range represents the minimum and maximum participant ROI median values.
(DOCX)

S7 Table Results based on log-linear least squares fitting for scan 1 and scan 2 single-participant ROI median and interquartile range (IQR) ultrashort-T_2_* values and between-scan absolute and percent change calculated from the two scan sessions for one participant. The results calculated using all seven TE values and the subset of three TE values are presented for all voxels and for only voxels with acceptable ultrashort-T_2_* fit (*R*^2^ ≥ 0.5). The median and interquartile range values are calculated for the sample of all voxels within each ROI.
(DOCX)

S8 Fig Bland-Altman plot for MnCl_2_ phantom ultrashort-T_2_* scan 1 versus scan 2. (TIF)

S9 Fig Bland-Altman plot for collagen phantom signal scan 1, orientation 1 versus scan 2, orientation 1. (TIF)

S10 Fig Bland-Altman plot for knee ultrashort-T_2_* scan 1 versus scan 2. Plots are shown for the values based on 7-TE and 3-TE and include all ultrashort-T2* values (all *R^2^*).
(TIF)

S11 Table Bland-Altman mean differences and 95% limits of agreement for scan-rescan measurements. Ultrashort-T_2_* results are for all ultrashort-T_2_* values (all *R^2^*).
(DOCX)
