# Correction: Ultrashort-T_2_* mapping at 7 tesla using an optimized pointwise encoding time reduction with radial acquisition (PETRA) sequence at standard and extended echo times

**DOI:** 10.1371/journal.pone.0346005

**Published:** 2026-03-27

**Authors:** Carly A. Lockard, Bruce M. Damon, Hacene Serrai

In the Ultrashort-T_2_* mapping subsection of Materials and methods. There is an error in the second sentence of the first paragraph. The correct sentence is: The MnCl_2_ phantom contained ten 5 mL centrifuge tubes having 0.03–30.50 mM MnCl_2_ tetrahydrate, based on target ultrashort-T_2_* values estimated using reported relaxivity relationships at 3T, and 30 mM NaCl in deionized water.

In Table 2, the column Phantom MnCl_2_ solution concentration [mM] is incorrect. Please see the correct Table 2 here.

In Fig 3A the values are incorrect. Please see the correct Fig 3 here.

In S2 and S3 Tables, the column Phantom MnCl_2_ solution concentration [mM] are incorrect. Please see the correct S2 and S3 Table here:

**Table 2:** Calculated ultrashort-T_2_* values, between-scan absolute and percent change, and ultrashort-T_2_* fit R^2^ from two scans for the MnCl_2_ phantom. The median and interquartile range values are calculated for the sample of all voxels within each ROI.

Phantom MnCl_2_ solution concentration [mM]	Scan 1 T_2_* median (interquartile range) [msec]	Scan 2 T_2_* median (interquartile range) [msec]	Between- scan T_2_* absolute change [msec]	Between- scan T_2_* percent change	Scan 1 mean *R*^*2*^	Scan 2 mean *R*^*2*^
30.50	0.31 (0.03)	0.31 (0.03)	0.00	1%	0.96	0.96
15.25	0.47 (0.04)	0.49 (0.05)	0.02	5%	0.98	0.95
10.16	0.78 (0.10)	0.78 (0.06)	0.00	0%	0.86	0.88
6.09	1.07 (0.20)	1.14 (0.20)	0.07	7%	0.97	0.95
4.06	3.55 (3.90)	2.03 (0.61)	1.52	43%	0.50	0.84
3.04	2.91 (2.87)	2.45 (0.68)	0.47	16%	0.54	0.73
2.02	5.12 (2.17)	6.61 (1.80)	1.49	29%	0.34	0.24
1.26	4.73 (0.73)	6.08 (2.19)	1.34	28%	0.37	0.30
1.21	3.96 (2.23)	4.32 (1.87)	0.36	9%	0.56	0.64
0.03	12.34 (9.61)	9.89 (10.40)	2.44	20%	0.08	0.11

**Fig 3:**
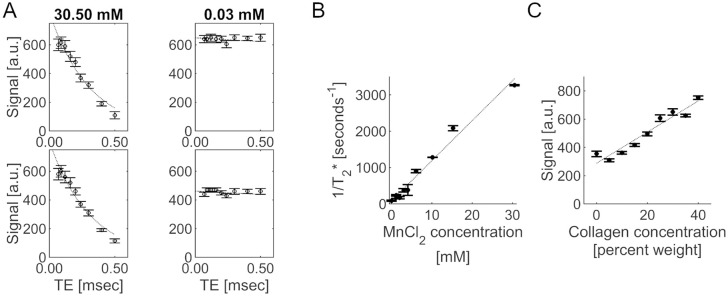
MnCl_2_ phantom plots. Plots showing examples of median measured (round markers) and fitted signal (dashed line) based on estimated ultrashort-T_2_* signal versus TE for three MnCl_2_ phantom solutions from two (top and bottom plots for each region) scans **(a)**, the relationship between mean 1/T_2_* and MnCl_2_ concentration, averaged over two scans **(b)**, the relationship between mean signal and collagen concentration at TE = 0.07 msec, averaged over three scans **(c)**. Error bars represent the interquartile range within each subregion for signal versus TE plots and the standard deviation between scans for the 1/T_2_* and signal versus concentration plots.

## Supporting information

S2 Table Results based on monoexponential fitting with a noise term for ultrashort-T_2_* values, between-scan absolute and percent change, and ultrashort-T_2_* fit *R^2^* from two scans for the MnCl_2_ phantom. (DOCX)

S3 Table Results based on log-linear least squares fitting for ultrashort-T_2_* values, between-scan absolute and percent change, and ultrashort-T2* fit *R^2^* from two scans for the MnCl_2_ phantom. (DOCX)
