# Supplementary material for: Ultrashort-T2* mapping at 7 tesla using an optimized pointwise encoding time reduction with radial acquisition (PETRA) sequence at standard and extended echo times
Source: PLoS One. 2025 Apr 17;20(4):e0310590. doi: 10.1371/journal.pone.0310590 (PMC12005508; doi:10.1371/journal.pone.0310590)
Supplement: S1 Table — (DOCX) [file pone.0310590.s001.docx]

**S1 Table. PETRA scan parameters selected as optimal for visualizing knee structures within a reasonable scan time.**

| Series type | Field of view (isotropic) [mm] | Matrix | Resolution (isotropic) [mm/pixel] | Radial views | Segments | Readout bandwidth [Hz] | TR [msec] | TE [msec] | Fat suppression | Scan time [minutes:seconds] |
| --- | --- | --- | --- | --- | --- | --- | --- | --- | --- | --- |
| High resolution for single-TE imaging | 170 | 496 | 0.34 | 50,000 | 20 | 160 | 7.07 | 0.07 | On | 6:55 |
| Low resolution for multiple-TE imaging | 170 | 112 | 1.52 | 70,000 | 40 | 397 | 4.21 | 0.07 to 0.58 | On | 5:20 at TE = 0.07 msec, 10:55 at TE = 0.58 msec |
