# Supplementary material for: Ultrashort-T2* mapping at 7 tesla using an optimized pointwise encoding time reduction with radial acquisition (PETRA) sequence at standard and extended echo times
Source: PLoS One. 2025 Apr 17;20(4):e0310590. doi: 10.1371/journal.pone.0310590 (PMC12005508; doi:10.1371/journal.pone.0310590)
Supplement: S4 Table — The results calculated using all seven TE values and the subset of three TE values are presented. The median value represents the median of the three median values (one per participant, for each ROI) calculated for each ROI. The range represents the minimum and maximum participant ROI median values. (DOCX) [file pone.0310590.s004.docx]

**S4 Table. Results based on monoexponential fitting with a noise term for knee tissue calculated median and range ultrashort-T_2_* values, ultrashort-T_2_* fit R^2^, and ultrashort-T_2_* fit X^2^ for single scans for three participants. The results calculated using all seven TE values and the subset of three TE values are presented.**

| Knee tissue | Median ultrashort-T_2_* (Range) [msec] | Median *R*^2^ (Range) | Median *X*^2^ (Range) | Median ultrashort-T_2_* (Range) [msec] | Median *R*^2^ (Range) | Median *X*^2^ (Range) |
| --- | --- | --- | --- | --- | --- | --- |
|  | Results when fitting to seven TE values | | | | | |
|  | All voxels | | | Voxels with *R*^2^ ≥0.5 | | |
| Cortical bone | 0.63 (0.21-1.34) | 0.93 (0.71-0.96) | 5.46 (3.59-6.36) | 0.60 (0.20-1.25) | 0.93 (0.82-0.96) | 4.26 (3.57-5.46) |
| Patellar tendon | 1.13 (1.02-1.54) | 0.91 (0.71-0.93) | 5.66 (3.43-18.24) | 1.02 (0.97-1.24) | 0.92 (0.81-0.94) | 5.06 (3.1-15.6) |
| Meniscus | 1.93 (1.73-2.27) | 0.59 (0.58-0.62) | 8.34 (7.97-8.94) | 1.75 (1.54-2.09) | 0.77 (0.73-0.79) | 6.10 (5.86-6.27) |
| Posterior cruciate ligament | 1.37 (1.22-2.25) | 0.79 (0.76-0.81) | 8.17 (3.46-10.29) | 1.30 (1.23-2.21) | 0.82 (0.81-0.84) | 7.60 (2.80-8.35) |
| Anterior cruciate ligament | 1.49 (0.96-1.93) | 0.71 (0.35-0.84) | 9.89 (7.51-11.26) | 1.29 (0.71-1.61) | 0.81 (0.77-0.9) | 5.46 (4.90-8.20) |
| Cartilage | 1.00 (0.87-1.19) | 0.88 (0.76-0.90) | 9.84 (6.44-13.76) | 0.91 (0.76-1.05) | 0.91 (0.86-0.93) | 6.58 (5.86-9.98) |
| Skin | 0.75 (0.71-0.92) | 0.98 (0.97-0.98) | 3.51 (2.10-4.41) | 0.75 (0.72-0.91) | 0.98 (0.97-0.98) | 3.46 (2.10-4.19) |
|  | Results when fitting to three TE values | | | | | |
|  | All voxels | | | Voxels with *R*^2^ ≥0.5 | | |
| Cortical bone | 0.75 (0.29-0.75) | 0.87 (0.83-0.97) | 1.05 (0.58-1.2) | 0.75 (0.29-0.75) | 0.87 (0.87-0.97) | 0.86 (0.58-1.05) |
| Patellar tendon | 1.01 (0.94-1.58) | 0.89 (0.52-0.93) | 1.03 (0.63-5.24) | 0.8 (0.80-0.96) | 0.97 (0.87-0.98) | 0.48 (0.35-2.98) |
| Meniscus | 0.91 (0.89-1.03) | 0.82 (0.76-0.88) | 1.98 (1.33-2.11) | 0.89 (0.80-1.00) | 0.88 (0.8-0.89) | 1.69 (1.22-1.76) |
| Posterior cruciate ligament | 0.76 (0.73-1.13) | 0.92 (0.49-0.93) | 1.53 (1.31-2.15) | 0.75 (0.72-1.00) | 0.92 (0.79-0.96) | 1.38 (0.70-1.45) |
| Anterior cruciate ligament | 0.79 (0.6-0.84) | 0.89 (0.56-0.98) | 1.55 (0.66-3.94) | 0.75 (0.52-0.82) | 0.92 (0.91-0.99) | 0.86 (0.27-1.16) |
| Cartilage | 0.71 (0.67-0.72) | 0.95 (0.93-0.96) | 0.89 (0.71-1.44) | 0.68 (0.66-0.7) | 0.96 (0.95-0.97) | 0.79 (0.59-1.11) |
| Skin | 0.74 (0.73-0.82) | 0.98 (0.98-0.99) | 0.42 (0.25-0.43) | 0.74 (0.72-0.82) | 0.98 (0.98-0.99) | 0.42 (0.25-0.43) |
