# Supplementary material for: Ultrashort-T2* mapping at 7 tesla using an optimized pointwise encoding time reduction with radial acquisition (PETRA) sequence at standard and extended echo times
Source: PLoS One. 2025 Apr 17;20(4):e0310590. doi: 10.1371/journal.pone.0310590 (PMC12005508; doi:10.1371/journal.pone.0310590)
Supplement: S6 Table — The results calculated using all seven TE values and the subset of three TE values are presented for all voxels and for only voxels with acceptable ultrashort-T2* fit (R2 ≥ 0.5). The median value represents the median of the three median values (one per participant, for each ROI) calculated for each ROI. The range represents the minimum and maximum participant ROI median values. (DOCX) [file pone.0310590.s006.docx]

**S6 Table. Results based on monoexponential fitting with a noise term for scan 1 and scan 2 single-participant ROI median and interquartile range (IQR) ultrashort-T_2_* values and between-scan absolute and percent change calculated from the two scan sessions for one participant. The results calculated using all seven TE values and the subset of three TE values are presented for all voxels and for only voxels with acceptable ultrashort-T_2_* fit (*R*^2^ ≥ 0.5).**

|  | Scan 1 median ultrashort-T_2_* (IQR) within each ROI [msec] | | Scan 2 median ultrashort-T_2_* (IQR) within each ROI [msec] | | Scan-rescan ultrashort-T_2_* absolute change [ms] | | Scan-rescan ultrashort-T_2_* percent change | |
| --- | --- | --- | --- | --- | --- | --- | --- | --- |
| Knee tissue | All voxels | Voxels with *R*^2^ ≥0.5 | All voxels | Voxels with *R*^2^ ≥0.5 | All voxels | Voxels with *R*^2^ ≥0.5 | All voxels | Voxels with *R*^2^ ≥0.5 |
|  | Results when fitting to seven TE values | | | | | | | |
| Cortical bone | 0.21 (0.19) | 0.21 (0.19) | 1.75 (1.32) | 1.69 (1.23) | 1.55 | 1.49 | 746% | 718% |
| Patellar tendon | 1.54 (1.45) | 1.24 (0.89) | 4.37 (265.60) | 1.39 (1.06) | 2.83 | 0.16 | 184% | 13% |
| Meniscus | 1.73 (1.30) | 1.54 (1.02) | 1.94 (1.37) | 1.71 (0.86) | 0.21 | 0.18 | 12% | 12% |
| Posterior cruciate ligament | 1.22 (0.73) | 1.23 (0.76) | 1.27 (0.80) | 1.24 (0.80) | 0.05 | 0.01 | 4% | 1% |
| Anterior cruciate ligament | 1.49 (1.07) | 1.29 (0.88) | 1.56 (0.93) | 1.34 (0.74) | 0.06 | 0.05 | 4% | 4% |
| Cartilage | 1.19 (1.17) | 1.05 (1.11) | 1.38 (1.06) | 1.15 (0.99) | 0.19 | 0.10 | 16% | 10% |
| Skin | 0.71 (0.28) | 0.72 (0.28) | 0.85 (0.33) | 0.85 (0.33) | 0.14 | 0.14 | 19% | 19% |
|  | Results when fitting to three TE values | | | | | | | |
| Cortical bone | 0.29 (0.31) | 0.29 (0.31) | 0.97 (0.41) | 0.74 (0.41) | 0.68 | 0.44 | 231% | 151% |
| Patellar tendon | 1.58 (2.57) | 0.96 (0.53) | 3.03 (8.62) | 1.08 (0.45) | 1.45 | 0.12 | 92% | 13% |
| Meniscus | 0.89 (0.48) | 0.80 (0.34) | 0.94 (0.38) | 0.86 (0.26) | 0.05 | 0.06 | 5% | 8% |
| Posterior cruciate ligament | 0.73 (0.33) | 0.72 (0.31) | 0.75 (0.27) | 0.74 (0.26) | 0.02 | 0.02 | 3% | 3% |
| Anterior cruciate ligament | 0.79 (0.29) | 0.75 (0.27) | 0.82 (0.25) | 0.79 (0.24) | 0.03 | 0.04 | 4% | 5% |
| Cartilage | 0.72 (0.48) | 0.68 (0.46) | 0.77 (0.34) | 0.74 (0.32) | 0.05 | 0.06 | 7% | 8% |
| Skin | 0.73 (0.22) | 0.72 (0.22) | 0.74 (0.20) | 0.74 (0.20) | 0.02 | 0.02 | 3% | 3% |
