# Supplementary figures and images for: Ultrashort-T2* mapping at 7 tesla using an optimized pointwise encoding time reduction with radial acquisition (PETRA) sequence at standard and extended echo times
Source: PLoS One. 2025 Apr 17;20(4):e0310590. doi: 10.1371/journal.pone.0310590 (PMC12005508; doi:10.1371/journal.pone.0310590)

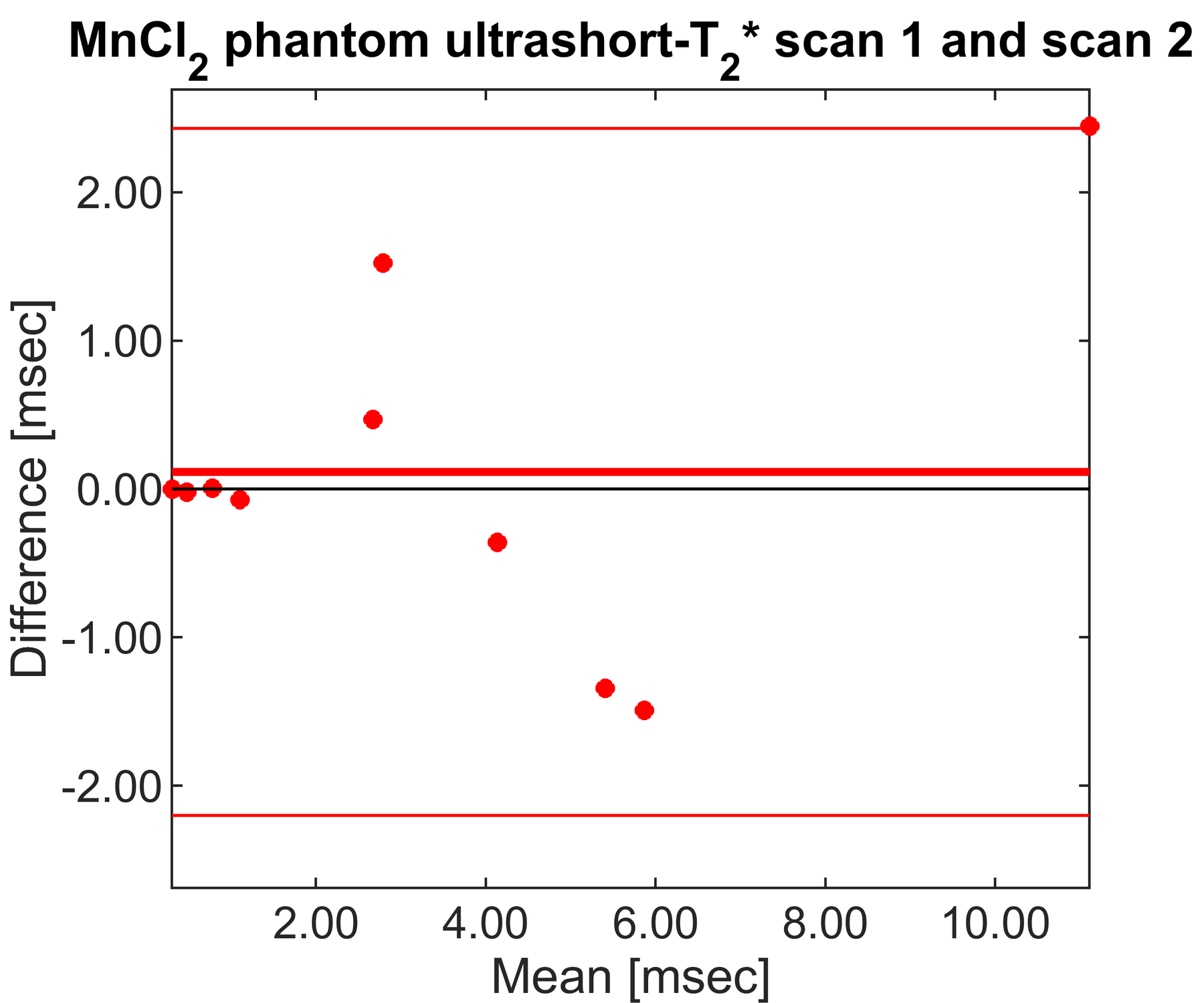

Supplement: S8 Fig — (TIF) [file pone.0310590.s008.tif]

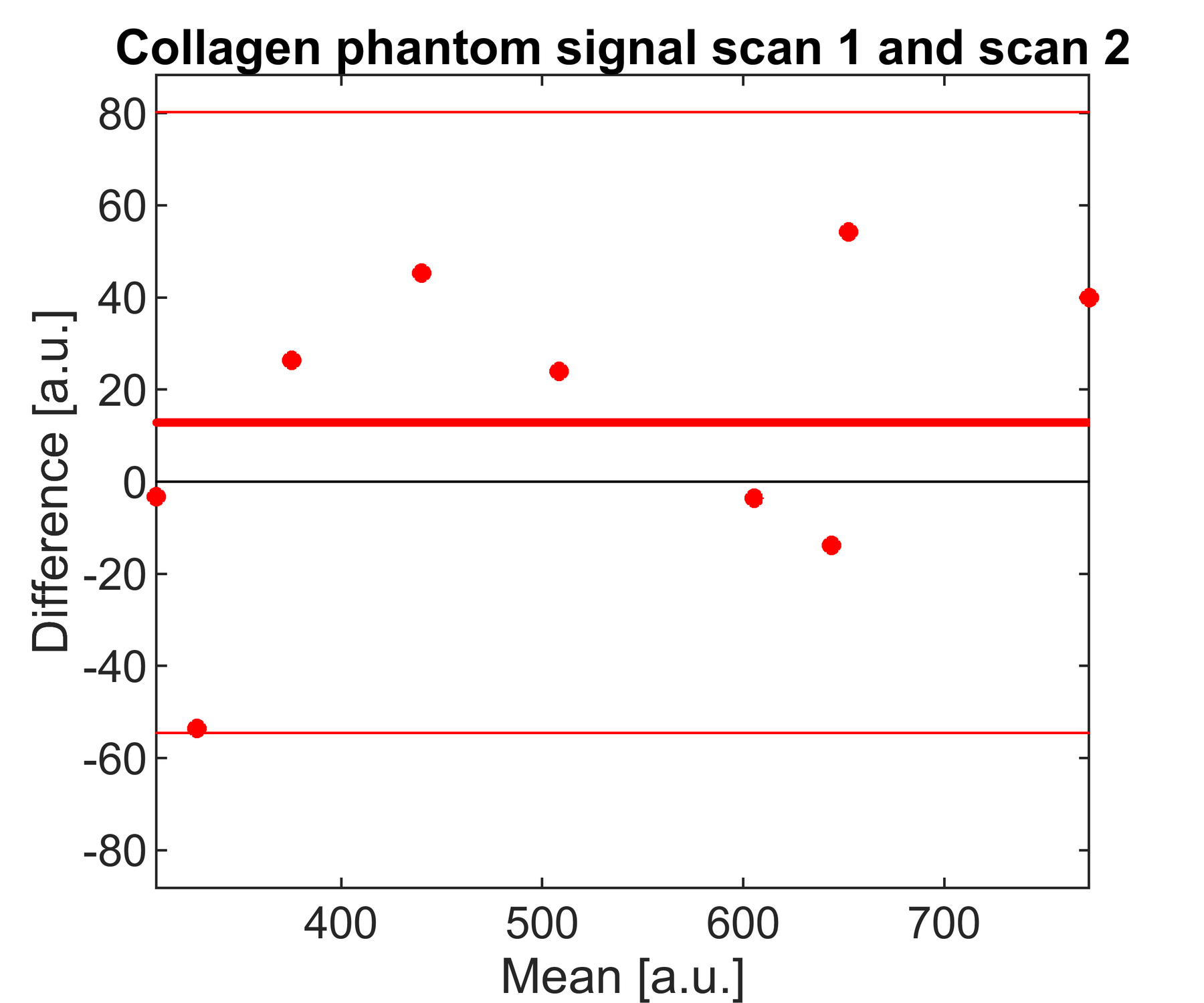

Supplement: S9 Fig — (TIF) [file pone.0310590.s009.tif]

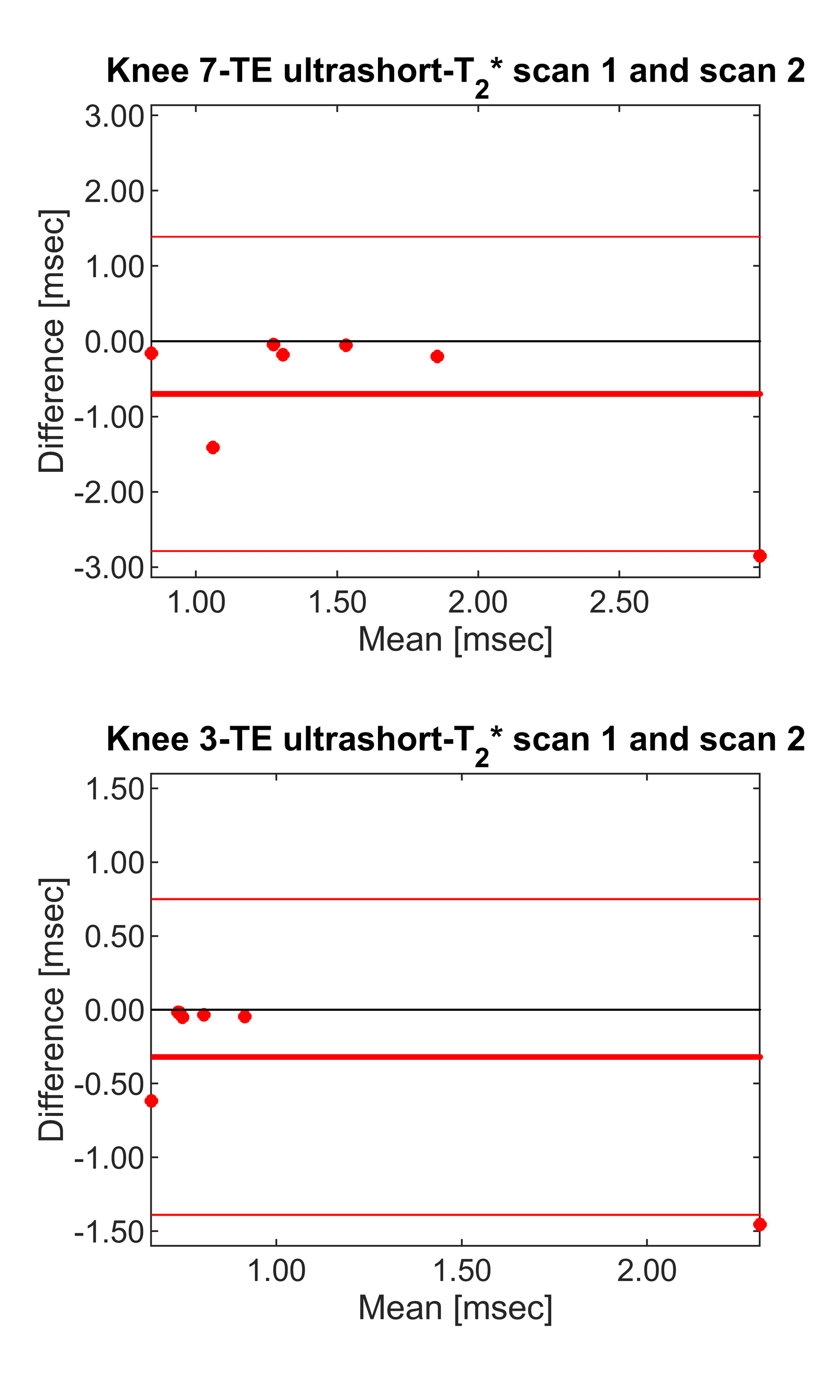

Supplement: S10 Fig — Plots are shown for the values based on 7-TE and 3-TE and include all ultrashort-T2* values (all R2). (TIF) [file pone.0310590.s010.tif]
