# Supplementary material for: Ultrashort-T2* mapping at 7 tesla using an optimized pointwise encoding time reduction with radial acquisition (PETRA) sequence at standard and extended echo times
Source: PLoS One. 2025 Apr 17;20(4):e0310590. doi: 10.1371/journal.pone.0310590 (PMC12005508; doi:10.1371/journal.pone.0310590)
Supplement: S11 Table — Ultrashort-T2* results are for all ultrashort-T2* values (all R2). (DOCX) [file pone.0310590.s011.docx]

**S11 Table. Bland-Altman mean differences and 95% limits of agreement for scan-rescan measurements. Ultrashort-T_2_* results are for all values (all R^2^).**

|  | Mean difference (95% limits of agreement) [msec] |
| --- | --- |
| MnCl_2_ ultrashort-T_2_* scan 1 versus scan 2 | 0.12 (-2.20, 2.43) |
| Knee tissue 7-TE ultrashort-T_2_* scan 1 versus scan 2 | -0.70 (-2.79, 1.39) |
| Knee tissue 3-TE ultrashort-T_2_* scan 1 versus scan 2 | -0.32 (-1.39, 0.75) |
|  | Mean difference (95% limits of agreement) [a.u.] |
| Collagen phantom signal scan 1, orientation 1 versus scan 2, orientation 1 | 12.84 (-54.54, 80.22) |
