# Supplementary material for: Correction: Ultrashort-T2* mapping at 7 tesla using an optimized pointwise encoding time reduction with radial acquisition (PETRA) sequence at standard and extended echo times
Source: PLoS One. 2026 Mar 27;21(3):e0346005. doi: 10.1371/journal.pone.0346005 (PMC13028329; doi:10.1371/journal.pone.0346005)
Supplement: S2 Table — (DOCX) [file pone.0346005.s001.docx]

**S2 Table. Results based on monoexponential fitting with a noise term for ultrashort-T_2_* values, between-scan absolute and percent change, and ultrashort-T_2_* fit *R*^2^ from two scans for the MnCl_2_ phantom.**

| Phantom MnCl_2_ solution concentration [mM] | Scan 1 T_2_* median (interquartile range) [msec] | Scan 2 T_2_* median (interquartile range) [msec] | Between- scan T_2_* absolute change [msec] | Between- scan T_2_* percent change | | Scan 1 mean *R^2^* | Scan 2 mean *R^2^* |
| --- | --- | --- | --- | --- | --- | --- | --- |
| 30.50 | 0.31 (0.03) | 0.31 (0.03) | 0.00 | | 1% | 0.96 | 0.96 |
| 15.25 | 0.47 (0.04) | 0.49 (0.05) | 0.02 | | 5% | 0.98 | 0.95 |
| 10.16 | 0.78 (0.10) | 0.78 (0.06) | 0.00 | | 0% | 0.86 | 0.88 |
| 6.09 | 1.00 (0.22) | 1.14 (0.21) | 0.14 | | 14% | 0.97 | 0.95 |
| 4.06 | 3.55 (3.90) | 2.03 (0.61) | 1.52 | | 43% | 0.50 | 0.84 |
| 3.04 | 2.91 (2.87) | 2.44 (0.68) | 0.47 | | 16% | 0.54 | 0.73 |
| 2.02 | 5.12 (2.18) | 6.61 (1.80) | 1.49 | | 29% | 0.34 | 0.24 |
| 1.26 | 4.73 (0.70) | 6.08 (2.19) | 1.34 | | 28% | 0.37 | 0.30 |
| 1.21 | 3.77 (2.14) | 4.32 (1.87) | 0.54 | | 14% | 0.56 | 0.64 |
| 0.03 | 12.43 (9.57) | 9.89 (10.40) | 2.54 | | 20% | 0.08 | 0.11 |
