# Supplementary material for: Correction: Ultrashort-T2* mapping at 7 tesla using an optimized pointwise encoding time reduction with radial acquisition (PETRA) sequence at standard and extended echo times
Source: PLoS One. 2026 Mar 27;21(3):e0346005. doi: 10.1371/journal.pone.0346005 (PMC13028329; doi:10.1371/journal.pone.0346005)
Supplement: S3 Table — (DOCX) [file pone.0346005.s002.docx]

**S3 Table. Results based on log-linear least squares fitting for ultrashort-T_2_* values, between-scan absolute and percent change, and ultrashort-T_2_* fit *R*^2^ from two scans for the MnCl_2_ phantom.**

| Phantom MnCl_2_ solution concentration [mM] | Scan 1 T_2_* median (interquartile range) [msec] | Scan 2 T_2_* median (interquartile range) [msec] | Between- scan T_2_* absolute change [msec] | Between- scan T_2_* percent change | | Scan 1 mean *R^2^* | Scan 2 mean *R^2^* |
| --- | --- | --- | --- | --- | --- | --- | --- |
| 30.50 | 0.25 (0.06) | 0.26 (0.05) | 0.01 | | 4% | 0.96 | 0.97 |
| 15.25 | 0.43 (0.05) | 0.45 (0.06) | 0.02 | | 5% | 0.98 | 0.97 |
| 10.16 | 0.72 (0.10) | 0.72 (0.07) | 0.01 | | 1% | 0.90 | 0.91 |
| 6.09 | 1.07 (0.20) | 1.11 (0.22) | 0.03 | | 3% | 0.97 | 0.95 |
| 4.06 | 3.42 (3.75) | 2.01 (0.64) | 1.41 | | 41% | 0.51 | 0.85 |
| 3.04 | 2.85 (2.70) | 2.45 (0.63) | 0.40 | | 14% | 0.54 | 0.74 |
| 2.02 | 5.05 (2.18) | 6.52 (1.91) | 1.47 | | 29% | 0.35 | 0.24 |
| 1.26 | 4.70 (0.71) | 6.08 (2.22) | 1.38 | | 29% | 0.38 | 0.30 |
| 1.21 | 3.97 (2.23) | 4.36 (1.96) | 0.39 | | 10% | 0.56 | 0.64 |
| 0.03 | 5.57 (37.64) | 7.25 (25.25) | 1.68 | | 30% | 0.13 | 0.14 |
